# Self-Learning Embedded System for Object Identification in Intelligent Infrastructure Sensors

**DOI:** 10.3390/s151129056

**Published:** 2015-11-17

**Authors:** Monica Villaverde, David Perez, Felix Moreno

**Affiliations:** Centre of Industrial Electronics (CEI), Technical University of Madrid; Jose Gutierrez Abascal, 6, 28006 Madrid, Spain; E-Mails: david.perez.daza@upm.es (D.P.); felix.moreno@upm.es (F.M.)

**Keywords:** embedded intelligence, sensors, cooperative sensor networks, object identification, self-learning

## Abstract

The emergence of new horizons in the field of travel assistant management leads to the development of cutting-edge systems focused on improving the existing ones. Moreover, new opportunities are being also presented since systems trend to be more reliable and autonomous. In this paper, a self-learning embedded system for object identification based on adaptive-cooperative dynamic approaches is presented for intelligent sensor’s infrastructures. The proposed system is able to detect and identify moving objects using a dynamic decision tree. Consequently, it combines machine learning algorithms and cooperative strategies in order to make the system more adaptive to changing environments. Therefore, the proposed system may be very useful for many applications like shadow tolls since several types of vehicles may be distinguished, parking optimization systems, improved traffic conditions systems, *etc*.

## 1. Introduction

The current state-of-the-art of the technology for increasing traffic safety fades the boundary between on-board and infrastructure safety systems. On-board -primary or secondary- safety systems focus on providing assistance to the driver or deploy all actions oriented to protect the occupants when an accident occurs. However, whereas primary safety systems try to avoid accidents, secondary safety systems aim to lessen the consequences for occupants. On the other hand, infrastructure safety systems are normally used to provide drivers with information concerning the environment which may change mainly due to weather conditions (rain, fog, snow, *etc.*), traffic conditions, state of the road, *etc*.

Additionally, the emergence of the Internet of Things (IoT) opens a new world of possibilities for the smart cities and traffic safety because new devices can take advantage of it to improve their functionality. The creation of a network composed by different objects connected each other is a cutting-edge concept. This is a new opportunity to enhance the city’s urban infrastructure since sensors can share information about their environment for handling different devices. This kind of information would be very useful to manage the traffic in a city and, at the same time, to increase the safety. 

Object identification is a very useful technique since it allows to distinguish different object types in order to act accordingly depending on the application requirements (e.g., object tracking to deal with the traffic density or object recognition to distinguish between vehicles and people for managing road light signals or to differentiate the vehicle type to handle toll.). However the detection of those objects is required before the identification. There are several sensors able to detect presence or motions; therefore there are multiple alternatives to carry this proposal out. Vehicle detection is not so complicated since vehicles have many metallic parts which may cause some variation of a magnetic field produced by some buried coils [1]. However, it is not suitable solution for people detection. Optical methods, like videocameras- based systems [2], can solve this problem since they are able to detect and to identify all kind of objects; but they are very computationally intensive and require expensive devices. On the other hand, ultrasonic sensors provide a short range, and hence they are not an extended solution. Nevertheless, despite the fact that radar technology is very similar to the previous one its range is larger since it uses an electromagnetic wave instead of an ultrasonic one. For all these issues, among all of the above mentioned alternatives, the radar technology is the one selected for this work. 

Radar sensors provide a signal when some motion occurs in theirs area of detection; therefore the object detection is not a complex task. However, object identification requires to analyze deeply the radar echo. For example, Fang *et al.* [3] utilized time-frequency analysis, multi-threshold detection, and Hough transform as main signal processing methods to extract speed and shape information of vehicles whereas Alaee and Amindavar [4] applied Chirplet transform to radar signal in order to make the target classification using the nearest neighbor clustering technique. Moreover, a two-stage support vector machine (SVM) classification method using Mel-Frequency Cepstrum coefficients is developed and proposed in [5]. Another alternative is proposed by Gharaibeh and Yaqot [6]. They presented a methodology based on Particle Swarm Optimization (PSO) that improved the computational speed for the nearest neighbor clustering technique. Other objects are analyzed in [7] where Briones *et al.* developed a radar system that operates in land and air and classifies different targets using an artificial neural network trained with the Levenberg-Marquardt algorithm. Our proposal implies to obtain information from the radar echo, in both time and frequency domains, in order to distinguish vehicles and pedestrian from other kind of objects. An expert system based on a classification tree [8] is developed to meet those requirements. Moreover, sensor cooperation is a support not only to guarantee high reliability but also to estimate the movement tracking of the detected object considering the devices which can detect that object. However, it is important to mention that the echo signal received from the same object type may be different whether some change in the environment is produced. For instance, if a branch of a tree interferes with the radar echo it may cause that a radar device, which was identifying pedestrian correctly, fails and therefore provides wrong identifications. Therefore, the system has also to be able to adapt to those changes using some machine learning techniques. 

The objective of this work is not only to identify different objects but also to adapt the system to changing environments combining system cooperation and machine learning techniques. Consequently, a generic proof of concept is presented which could be employed for different applications such as automatic traffic management or effective control for shadow tolls. 

In many situations, traffic jams may be avoided by implementing a suitable traffic lights operation management in a city. For instance, nowadays, a simple solution is provided in some crossings to deal with the traffic light control. It consists of pushing a button when a pedestrian has the intention to cross a road. However, this is not an automatic solution and also it is not useful for all people (e.g., blind people) because it requires pressing the button to turn on green the pedestrian signal. Using the proposed radar-based system would be possible to automate this process, especially at night or low pedestrian density streets. When a radar-based device identifies a pedestrian, it can communicate to other devices that there is a person on the street which causes an effective network between this radar-based device and the other ones located closely. The network goal is not only to pass information about the presence of a pedestrian in order to locate him/her, but also to verify that the identification of that radar device is correct (hit). This verification can be done through cooperation since the network gives the opportunity make a joint decision using multiple hypotheses about the object type. If a radar device provides ongoing misses (wrong identifications), it should modify its own identification algorithm in order to adapt its behavior to this new situation.

On the other hand, as mentioned before, this system can also be useful for shadow tolls since it may distinguish several vehicles types such as cars, trucks or motorcycles in order to pay the tax accordingly. It could minimize the contractual payment made by a government per driver to a private company since different fares should be applied depending on the type of the vehicle.

Moreover, the proposed system is very versatile since it can be supported by the city infrastructure, as previous examples demonstrate, but also it can be applied as a part of a vehicle in order to improve vehicle-to-vehicle communication. It means that each vehicle can be dealt as a device inside the cooperative network and it can share information about the presences in the environment to others vehicles at real time. Therefore, the autonomous driving could be enhanced since each vehicle can share environment information while it is moving around.

All these issues make the proposed system extremely useful for short-term outlooks on road safety applications. However, one of the most important requirements of this work is to implement all of these functionalities in an embedded system. In this way, an external computer is not required since a compact hardware platform can be integrated in multiples system to provide it with these functionalities.

The rest of the article is organized as following. Section 2 specifies the system implementation and the general proposed scheme. In Section 3 some issues about theoretical concepts of decision tree and clustering are given, but alsoand the cooperative network and some cooperative strategies are presented. Section 4 shows the real and simulated results. And finally, in Section 5, some conclusions and future works are provided.

## 2. Overall System

In order to develop the proposed system, a low-cost hardware platform was implemented [8]. This is based on a DSP (Digital Signal Processor) which is in charge of analyzing the radar signal and implementing the expert system that deals with the radar information to identify the detected object. A low-cost and low-power radar device has been selected which works in the 10 GHz band (ISM band) so no authorization is required for its use. 

The expert system is based on a decision tree therefore it has to be trained before the commissioning. A decision tree is a flowchart-like probabilistic method for classifying different entities analyzing some of their intrinsic parameters. They are supervised methods within the machine learning field so they require a train stage which implies to create the structure of the tree according to some real examples. In this work a decision tree is used to classify different kind of objects such as pedestrians, vehicles or other types of road-environment objects and, in our case under study, the previous training consists of collecting several echoes of the radar device provided by different kind of detected objects. After that, these signals are used to extract the main characteristics of each object category so every new taken signal will correspond to a specific category. Consequently, the proposed device –or node- is composed by the radar, a conditioned stage for adapting the radar signal, a DSP for signal processing and some communication interfaces.

In spite of the fact that this radar-based device can detect and identify different objects, which are previously defined, our new proposal for this work tries to go further. Adaptation to changing environment and also reliability improvements are sought; therefore some modifications of the early device are required. The new proposal implies to modify the structure of the decision tree in order to allow internal changes and also it can include some cooperative skills using a network support. 

**Figure 1:**
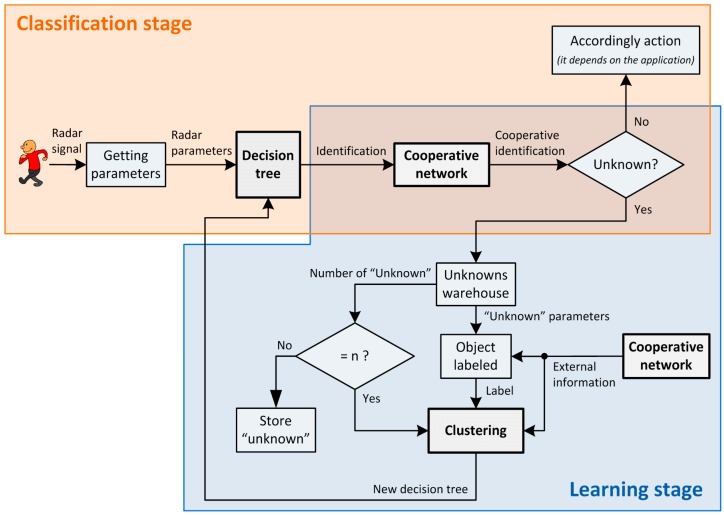
Whole scheme of the proposed system.

On the one hand, cooperation will provide more reliability since it allows make a joint decision taking into account different hypothesis over the same object. Moreover, on the other hand, a dynamic classification tree is proposed in order to adapt the system behavior to some environment changes using also the cooperative network and some clustering techniques. The whole scheme of the proposed system is shown in Figure 1 where two different stages are defined. 

The classification stage provides a category which corresponds to the kind of the detected object. The main functionality of this stage is supported by the classification tree- or decision tree- since it has to provide the identification of the new detected object according to its previous training. However, the decision tree has been modified for including an “unknown” category. This category collects the objects which cannot be classified with a high level of reliability. Furthermore, although the decision tree provides a valid category, the final decision is not only given by this classification tree but also the cooperation among the others has to be taken into account. Therefore, a communication network is required to provide the support for sharing information among different devices placed at different locations but belonging to the same network. Once a classification tree provides an identification result, this has to be shared among the rest of the nodes that belong to the same cooperative network in order to obtain a cooperative identification result. Using this procedure, the system is able to assure a more reliable identification since different nodes participate in the final decision. 

**Figure 2:**
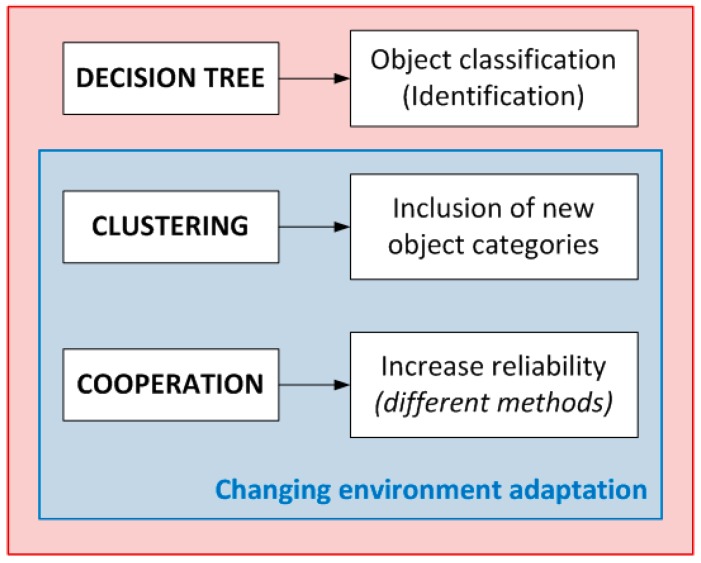
Identification process combining different machine learning methods and cooperation.

The adaptation to changing environments is achieved in the learning stage. In this process the system uses those objects that have been classified as “unknown objects” for creating a new label to define them correctly. In order to assign the label, cooperative contributions have to be taken into account so the cooperative network is also in charge of defining this new label. Figure 2 specifies the contribution of the cooperation and machine learning methods to the whole system functionality.

## 3. Materials and Methods

### 3.1. Decision Tree for Implementing the Expert System

As mentioned before, the initial classification tree has suffered some modifications to provide a new category to collect the identifications of new unknown objects. Those modifications are based on pruning methods but also clustering techniques have to be applied.

Decision tree algorithms are supervised methods for classification. They are based on performing training set partitions recursively until the whole training set is perfectly classifiable. However, the performance of the algorithm is not the only feature to be considered since each application may require its own specifications; therefore machine learning algorithms have to be adapted to a particular application. For instance, Mantas & Abellán [9] proposed a modification of the C4.5 algorithm for building a decision tree able to classify noisy data which belong to a low reliable training set. C4.5 algorithm is a method proposed by Quinlan [10] which, at the same time, is a modification of the previous ID3 proposed by the same author in 1986 [11]. ID3 uses two metrics to select the feature which performs the best splitting of the training set: a measure of the amount of uncertainty for a given dataset, as it called entropy E(S) and a measure of how much the uncertainty was reduced after splitting the dataset by a feature, as it called information gain, IG(S, F). Both formulas are given by Equations (1) and (2) respectively, where S is the dataset, F is the feature, p_i_ is the likelihood of appearance of each class in S and p_v_ is the likelihood of appearance of the partition in the S.

(1) $$E(S)=-\sum_{i=1}^{No.\;of\;classes}{p_{i}\cdot \log}_{2}\left(p_{i}\right)$$

(2) $$IG(S,F)=E(S)-\sum_{v\text{​ }\in \text{​ }Values}p_{v}\cdot E(S_{v})$$

On the other hand, C4.5 uses a ratio in order to prevent errors in datasets with very different feature scales. The ratio used to select the best feature is the gain ratio GR(S, F) and it is calculated as Equation (3) says.

(3) $$GR(S,F)=\frac{IG(S,F)}{E(S_{v})}$$

C4.5 uses a post-pruning method to generate the decision tree which implies creation and storing of the whole tree in order to prune it at the end; therefore the required memory for post-pruning is too large. Additionally, C4.5 computes the entropy in a variable number of points depending of the training set used which involves a big computational cost.

The decision tree algorithm proposed in this work is an adaptation of the original C4.5 algorithm. It is based on replacing the variable number of points of the computed entropy by a constant number of points in order to achieve a reduction in the computational cost. Moreover, the post-pruning method is replaced by a pre-pruning one for reducing the memory requirements.

**Figure 3:**
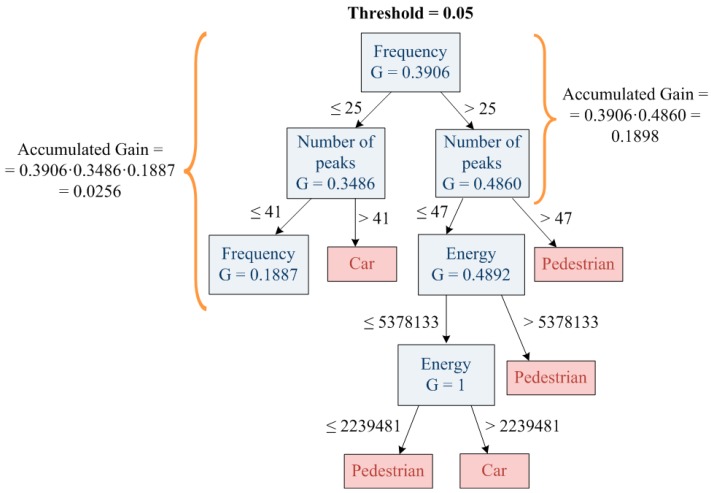
Pruning process.

Additionally, pre-pruning is in charge of providing the system with self-learning capabilities, *i.e.*, the decision tree is created from the top to the bottom and for every node of the decision tree, the gain ratio (GR) is computed. The cumulative product of the gain ratio (GR) of all the upper decision tree levels is called accumulated gain (AG). If the accumulated gain (AG) is greater than the threshold (pruning threshold), the decision tree will continue growing. In other case, the decision tree will be pruned (Figure 3).

In a regular decision tree algorithm, the pruned branch will be replaced by a leaf that represents the majority of cases of the pruned branch. However, the proposed algorithm replaces this branch by the “unknown” class. The “unknown” class collects all the objects with low gain ratios, hence, unusual object or those which are outside of the training set. Due to the fact that these objects do not fit in the training set, they must be different objects to the trained one (car, pedestrian or environment) thus they have to be learned. Consequently, the ‘unknown’ class is the base of the self-learning capability.

### 3.2. Clustering

Clustering algorithms are unsupervised methods for classification. They are based on creating clusters within the training set gathering the instances that are closest to each other in an n-dimensional space formed with the training set features. In this work, clustering techniques are used to make groups of unknown objects in order to create a new category for modifying the decision tree internal structure (the “unknown” classes).

The most popular clustering algorithm is *k-means* which is widely used since 1967 when MacQueen [12] implemented it for the first time. This algorithm needs two parameters to be executed: the number of clusters searched and their centroid. 

There are many works which define different adaptations of this algorithm improving some of its characteristics. The algorithm initialization is a common issue to be enhanced [13], but other modifications are also possible [14]. According to these lines, an adaptation of *k-means* algorithm is proposed in this work for reducing its memory usage and its computational complexity to adapt it to a non-complex DSP. 

**Figure 4:**
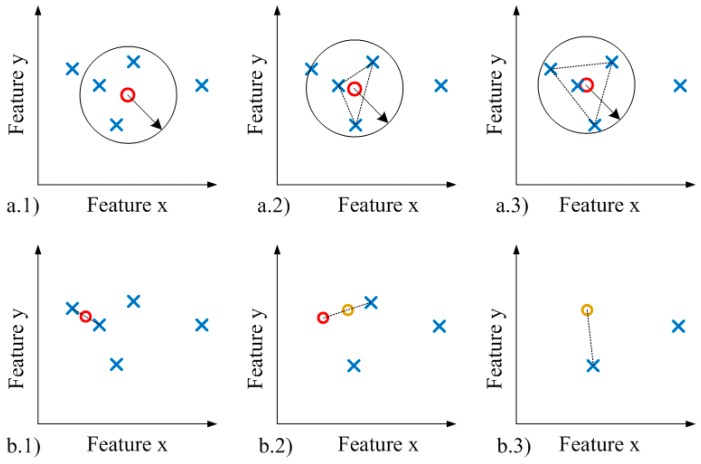
(**a**) Centroid-based method; (**b**) Distance-based method.

The proposed adaptation consists of replacing the centroid-based clustering by a distance-based one. *K-means* starts from the given centroid (Figure 4a(1)) and computes the new centroid taking into account the instances inside of its influence (Figure 4a(2)). The process is executed iteratively until the computed centroid is almost stable (Figure 4a(3)). In contrast, the distance-based adaptation uses the two nearest instances to extract the midpoint (Figure 4b(1)). Once the midpoint is found, it is used to calculate a new midpoint by taking its nearest instance (Figure 4b(2)). This process is executed iteratively until the distances between the nearest instances/clusters are larger than a predefined threshold (clustering threshold) (Figure 4b(3)).

### 3.3. Cooperative Network

Cooperative models can be used to create more powerful systems by using information from different subsystems. These models are used to reduce the complexity of a system since they allow to distribute different tasks in different subsystems. Otherwise, they can also be used as an alternative to enhance the reliability of the system since several devices work together to reduce the unawareness of the system giving better solutions. 

In this work, the cooperative model is applied in order to improve the reliability of the identification process and also to enhance the system adaptation (Figure 1). On the one hand, if the system is able to increase the success rate of the object identification it is considered more reliable since the number of wrong identifications is reduced. Therefore, one of the main ideas of this work is to manage information provided by different radar devices which are placed in different locations. In this way, it is possible to obtain different points of view of the same object which permits to manage more information to distinguish the kind of the object. On the other hand, this information, provided by the cooperative model, can be useful to label the “unknown” objects when a subsystem is not able to provide a specific category with a high level of reliability. This procedure allows to change the internal structure of the decision tree according to the received information given by other devices just in case of one of them does not offer a category. Thereby every device can operate in different environments and may also be more competent as their knowledge base is updated. In others words, a learning process is proposed for adapting the system to changing environments considering the information that all devices share among them using the cooperative network.

#### 3.3.1. Network Implementation

In this work a wireless network has been selected as a communication support since it offers more flexibility, scalability and simplicity of installation than a wired one, especially considering that it is not known *a priori* the number of devices which are going to be connected to that network. Furthermore, ZigBee is the selected technology because it offers a high degree of versatility when the network topology is created, it has a very low consumption and also offers a bandwidth according to the level of the data required for the transmissions. In particular, the selected module -ETRX2HR-PA- includes a power amplifier for transmissions and a U.FL connector for connecting an external antenna which allows to increase the gain. Also, this module implements the ZigBee protocol and it is possible to control it using AT commands. Thereby, for this work an external antenna is connected to increase the communication range. This antenna has a low gain which, however, is enough for our experiments. In any case, it can be replaced by another one according to the application requirements.

Despite of the advantages of wireless communications, the node coverage is an important consideration in this kind of networks since the communication among nodes has to be ensured avoiding isolated nodes. In order to determine the range suitable between the devices for guarantying the communication, a maximum distances analysis has been made applying the Friis Equation (4).

(4) $$\frac{P_{R}}{P_{T}}={\left(\frac{\lambda }{4\cdot \text{Π}\cdot D}\right)}^{2}\cdot G_{T}\cdot G_{R}\cdot L_{T}\cdot L_{R}\cdot L_{OBS}$$

**Figure 5:**
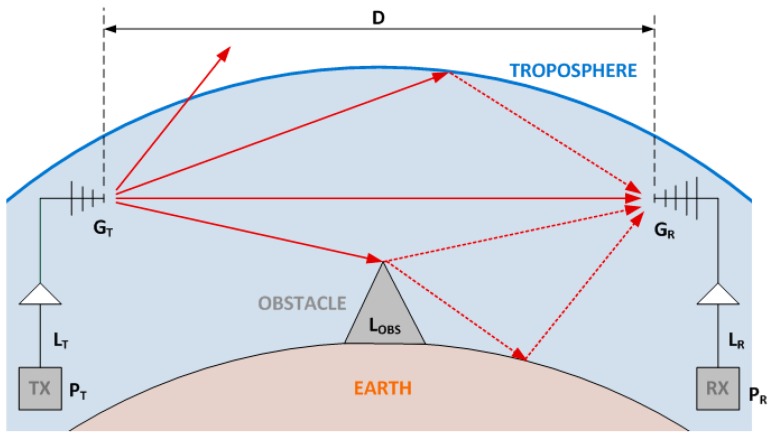
Signal transmission model in an outdoor environment with obstacles.

The Friis formula allows to estimate the received power always when the transmitted power (P_T_), the gains (G_T_ and G_R_), the distance between the antennas (D) and the losses (L_T_, L_R_ and L_OBS_) are known. Figure 5 shows the signal propagation model for outdoors scenarios and illustrates how the sending signal is propagated in any direction and the received antenna receives direct and bounced signals. However, as that figure shown, part of the signal does not reach the received antenna. Therefore, the Friis formula is useful to calculate how far the nodes can be placed to ensure reliable links. 

#### 3.3.2. Cooperative Decisions

There are multiples ways to obtain the cooperative solution. In this work several alternatives are proposed, however each one has different properties and requires its own network topology. Thanks to ZigBee properties it is possible to create different connections among devices. However, in any case, a coordinator is required not only to create the network but also to manage the final solution of the system. Therefore, in terms of functionality, the coordinator is in charge of taking the final identification result so it requires collecting information from other devices to give this solution. 

**Figure 6:**
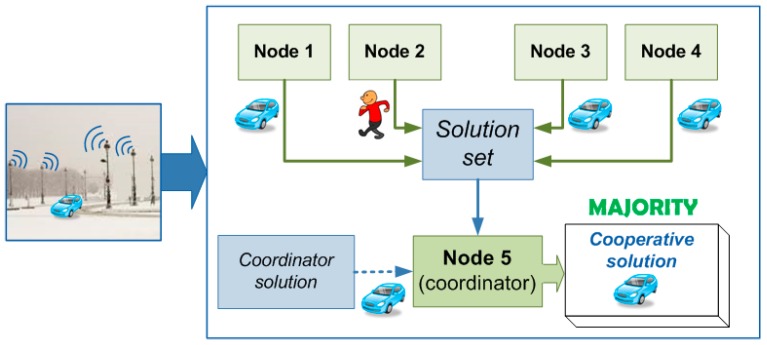
Majority-based voting.

The majority voting is the simplest procedure but it has no learning properties due to the fact that it does not use any mechanism for considering past experiences (Figure 6). In this case, the coordinator collects all the identifications given by the other devices and analyzes which is the most voted category that will be the final identification for the detected object. Therefore, this procedure requires a star topology since just the coordinator has to be connected with the other nodes. However, ZigBee allows to create a mesh topology in which every device is connected with the rest. This advantage is useful in case the coordinator falls down since other device can take possession of the coordinator role.

**Figure 7:**
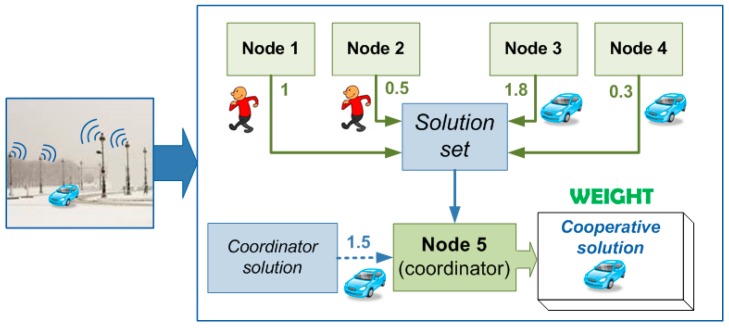
General weight-based voting.

On the other hand, weighting-based procedures provide a way to acquire learning since these methodologies consist of assigning a value to every single system in order to weight their contributions (Figure 7).

Different weighting procedures were analyzed in [15] such as global weighting and conditional weighting. In the first one, each device has its own assigned weight which provides information on how good each device is independently of the category of the detected object. After detection, each device will be rewarded if its identification matches with the cooperation result, otherwise it will be penalized. The reward consists of incrementing its weight whereas the penalty consists of decrementing it. The increment or the decrement is constant and it indicates how much the weights between two detections should be amended. In contrast, conditioned weighting-based voting allows to consider the kind of the detected object for weight’s modifications. Therefore, more than one weight is required for every device instead just one. It implies more complex management of weights since the values which has to be modified depend not only on the identification given by the device but also on the type of the cooperative solution. It means that the procedure of the weight’s modification is different depending on the category of the cooperative solution and whether or not each partial identification matches that solution. 

Nevertheless for both proposed voting procedures, increments and decrements are given by a constant value defined at programming time. In order to avoid these dependencies and to isolate the programing phase and the run-time phase, in [16] other alternatives for weights modification were proposed. These new methodologies search better adaptation to changing environments since the new goal is to adjust the variations of the weights according to the system behavior. Therefore, weights should be modified in correspondence to the system requirements instead of using a fixed variation. Two different alternatives were proposed but any of them are still implemented in the real system. On the one hand, the first approach allows to assign weights for every device applying a particle filter. A particle filter is a Montecarlo-based method suitable for predicting future states of non-linear systems with non-Gaussian noise. It is based on a set of samples of the state where each individual sample is called particle. These particles are weighted according to the real measure of the state in order to estimate the future state of the system. On the other hand, another method was proposed for evaluating the probability of success of all subsystems in order to assign an appropriate value to each one of them.

**Figure 8:**
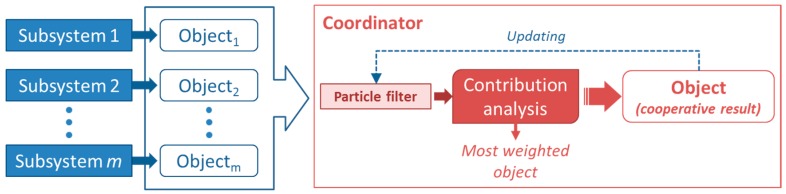
Particle filter implementation made by the coordinator.

In the first proposed approach, which uses the particle filter-based method, particles should represent possible combinations of the assigned weights for every device and they has to be placed randomly in the *n-dimensional* space (where *n* is defined by the number of devices), therefore each particle has *n* components. After several iterations, a particles’ convergence area should be achieved and this will mark where the best combinations of weights are located considering the previous behavior of the system. Once an object is detected, each device can either send its own identification result to the coordinator or share it with the rest in order to provide a set of weighted partial identification results. In both cases a coordinator is required to collect the results provided by the subsystems. However, in the first case, every device generates only its own partial result and the coordinator applies the cooperation through weighting all of those results using the particle filter concept (Figure 8).

**Figure 9:**
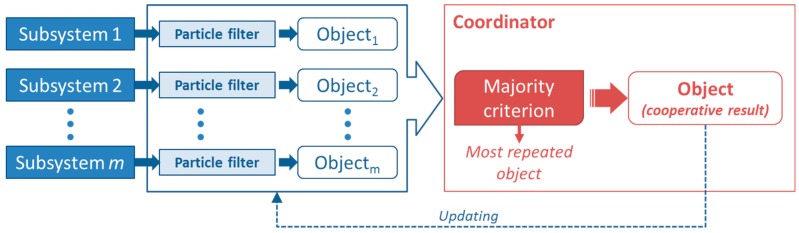
Particle filter implementation made by the subsystems.

In contrast, in the other case, each device has to analyze every partial result using its own particle filter to produce a partial weighted solution dependent on the rest of identifications, so it requires to share information not only with the coordinator but also with the rest of them (Figure 9).

**Figure 10:**
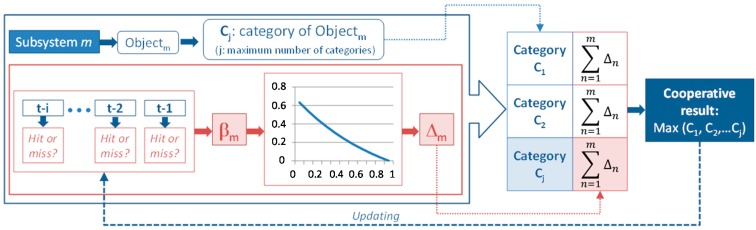
Procedure based on the last i-evolutions.

The other proposed alternative has nothing to do with particle filter. In this case, the probability of success considering the last *i*-evolutions must be analyzed. If one device has a large probability of success, and consequently small probability of failure, its contribution has to be greater than the one provided by the others with lower success probability (Figure 10). 

This method implies to be continuously updating the hit rate of each subsystem taking into account the last i-evolutions. The result provided by each subsystem has to be weighed and its partial contribution will be adding to the relevant category. The contribution of every device is given by Equation (5) where Δ_m_ is the contribution added by the device *m* and β_m_ is its probability of failure considering the last *i-*evolutions. The term e^−1^ has to be subtracted due to the fact that we want to cancel the contribution of those devices which did not have any hit in the last *i*-evolutions.

(5) $${\text{Δ}}_{m}=\;e^{-\beta_{m}}-\;e^{-1}$$

## 4. Results

In this section several results from different points of view are presented. First of all, the coverage analysis shows how far the devices can be placed to guarantee communication among them avoiding isolated devices. Moreover, some simulated results related to machine learning algorithms are also presented. However, in order to achieve an interpretable view of those results, graphs presented in this section show only a representation in two or three dimensions, hence in some graphs the less significant features of the training set are not observed. Finally, the system performance is analyzed to demonstrate if the proposed solution is suitable for embedded systems.

Nevertheless, the system cannot be tested completely since the cooperation has not been included yet. Therefore, the whole system does not label the “unknown” objects until now. However, the presented results are enough to evaluate in general terms whether or not the proposed architecture could be appropriated.

### 4.1. Coverage Analysis

The parameters that have been considered for the coverage analysis are shown in Table 1. It is important to mention that the obstacle losses have been estimated for a multiobject space according to the recommendations given by the ITU (International Telecommunication Union).

**Table 1:** Parameters for the coverage analysis.

Parameter	Value	Value (Natural Units)
Frecuency (f)	-	2.4 GHz
Wave length (λ)	-	0.125 m
Transmitted power (P_T_)	3 dBm = −27 dB	1.995 mW
Antenna gain (G_T_ y G_R_)	32.39 dBm = 2.3880 dB	1.7330
Obstacle losses (L_OBS_)	0 dBm = −30 dB	0.001
Cable losses (L_T_ y L_R_)	29.77 dBm = −0.2325 dB	0.9479
Sensitivity	−97 dBm = −127 dB	0.1995 pW

**Figure 11:**
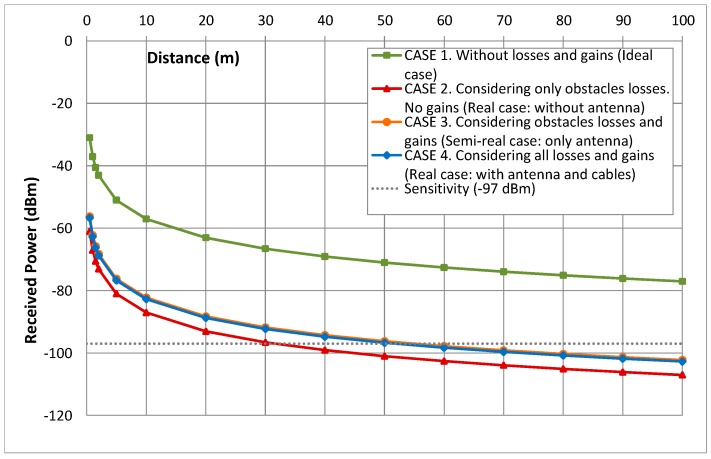
Estimation of maximum distances using Friis formula.

**Figure 12:**
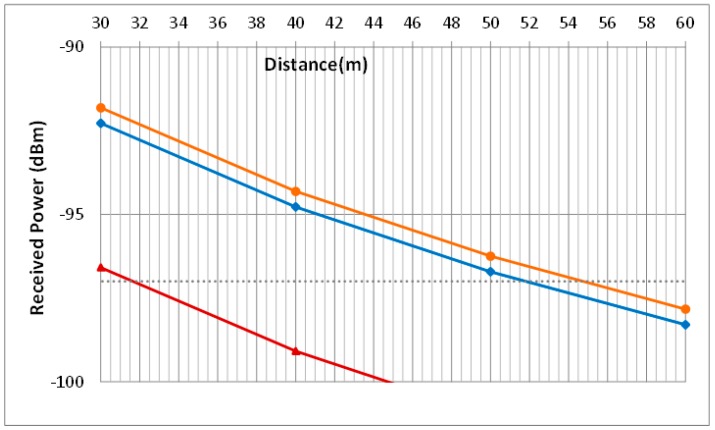
Zoom of maximum distances estimations.

The coverage analysis has been studied for different cases. Figure 11 and Figure 12 show the theoretical results obtained when the Friis Equation (4) is used and the parameters of the Table 1 are applied.

These results demonstrate that the cable losses are almost irrelevant since cases 3 and 4 are very similar. Furthermore, simulations show that the maximum distance among devices could be more than 50 m since the receiver sensitivity is −97 dBm. However, it is advisable to give a security margin and no to go so closely to the sensitivity value. Therefore, our real measures verify that 35 m is an appropriated distance to assure nodes coverage.

### 4.2. Decision Tree Pruning and Clustering Results

Results shown in this section do not consider the cooperative model implementation. It means that the modification of the decision tree is made using only the information generates in the own device without using external information provided by the cooperative network. The inclusion of the cooperative information for modifying the decision tree is one of the future works proposed in this article. Therefore, in this section the influence of both pruning and clustering thresholds are evaluated.

**Figure 13:**
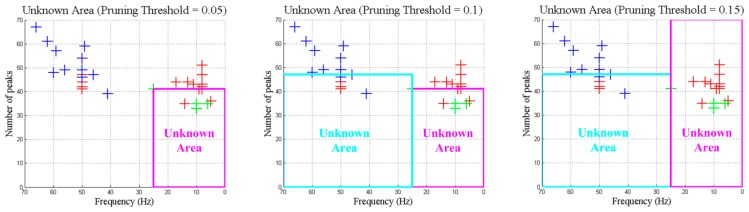
Evolution of “Unknown Area”.

In order to analyze the influence of the pruning in the decision tree algorithm, different pruning thresholds have been simulated. Results are shown in a graph that represents the training set and the unknown area created during the pruning process. In the case of a pruning threshold of 0.05, the created “Unknown Area” is small compared to the total area available to accommodate new objects. In contrast, a pruning threshold of 0.15 generates the “Unknown Area” to be too large causing some classes to disappear. Figure 13 shows the evolution of the “Unknown Area” with the increasing of the pruning threshold.

**Figure 14:**
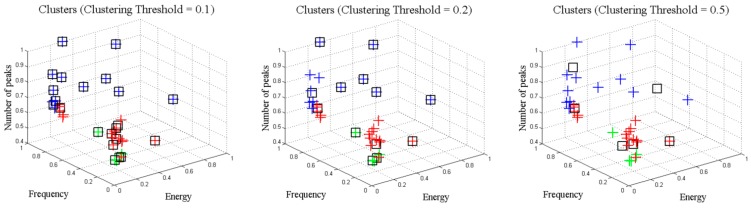
Evolution of clusters.

Identically to the decision tree algorithm results, the influence of the clustering threshold has also been analyzed. Therefore, different situations have been simulated using different clustering thresholds. When a clustering threshold of 0.1 is applied, the number of clusters generated is high and the problem is that most of the clusters are unitary, as the clusters are not a combination of several instances. In contrast, a clustering threshold of 0.5 creates heterogeneous clusters where the instances are too different among them. Figure 14 and Table 2 show the evolution of the clusters for different threshold values.

**Table 2:** Clustering threshold.

	Clustering Threshold		
	0.1	0.2	0.5
**Number of Groups**	22	13	6
**Unitary Groups**	17 (77.27%)	9 (69.23%)	1 (16.67%)

**Figure 15:**
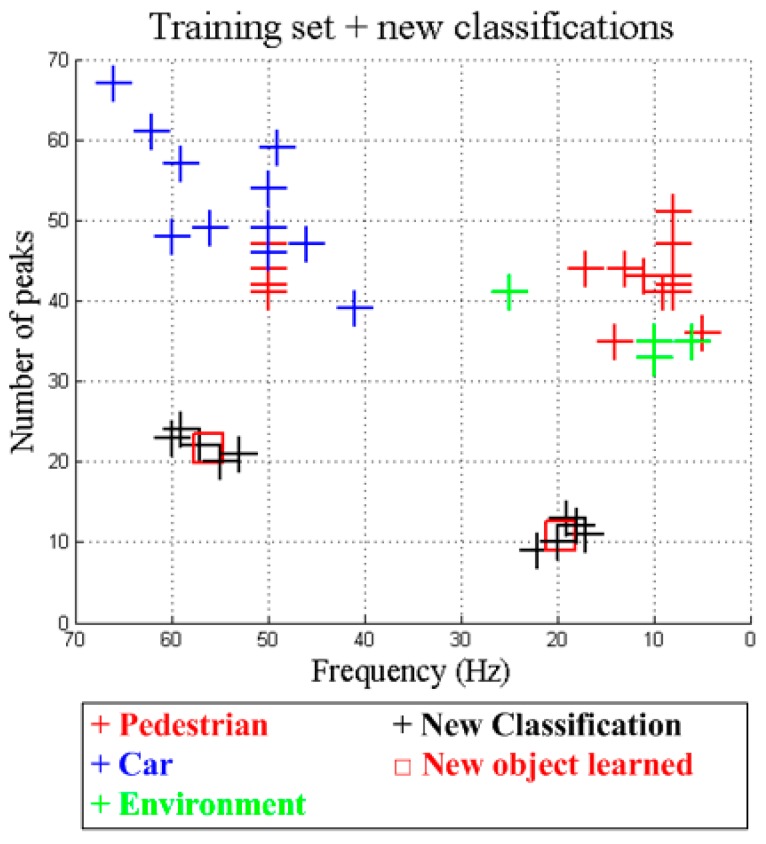
Training set including new classifications.

The validation of the dynamic decision tree learning architecture was developed in two different stages. During the first stage, a simulation of 10 new classifications was carried out. The result of the learned instances corresponds to 2 different objects. In Figure 15 the obtained results are presented.

**Figure 16:**
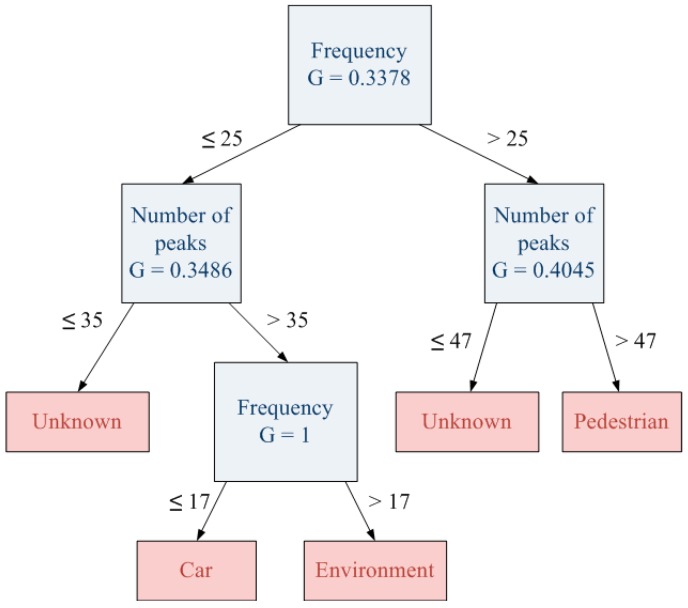
New decision tree generated.

In spited of the fact that new object has been identified, the new decision tree generated after the completion of the clustering process does not include any of these new learned objects. This effect is produced since the decision tree does not have enough information to include new objects in the decision tree. Figure 16 shows this new decision tree which does not include any “unknown” category.

**Figure 17:**
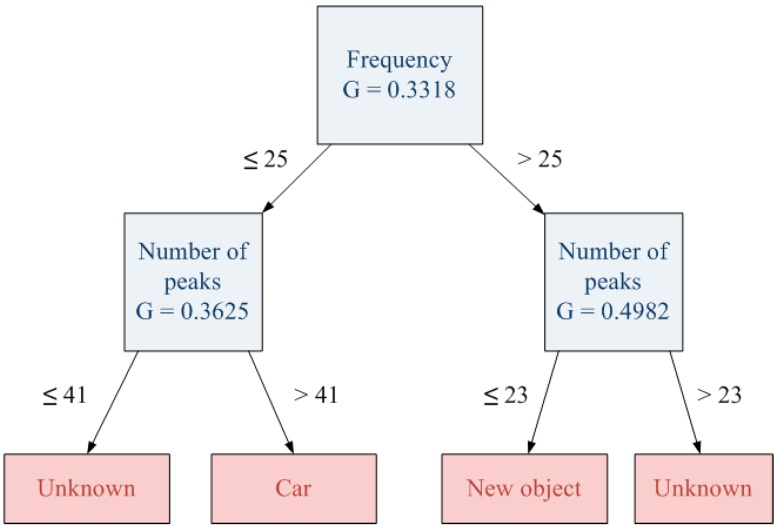
Decision tree with the same pruning threshold (0.1).

**Figure 18:**
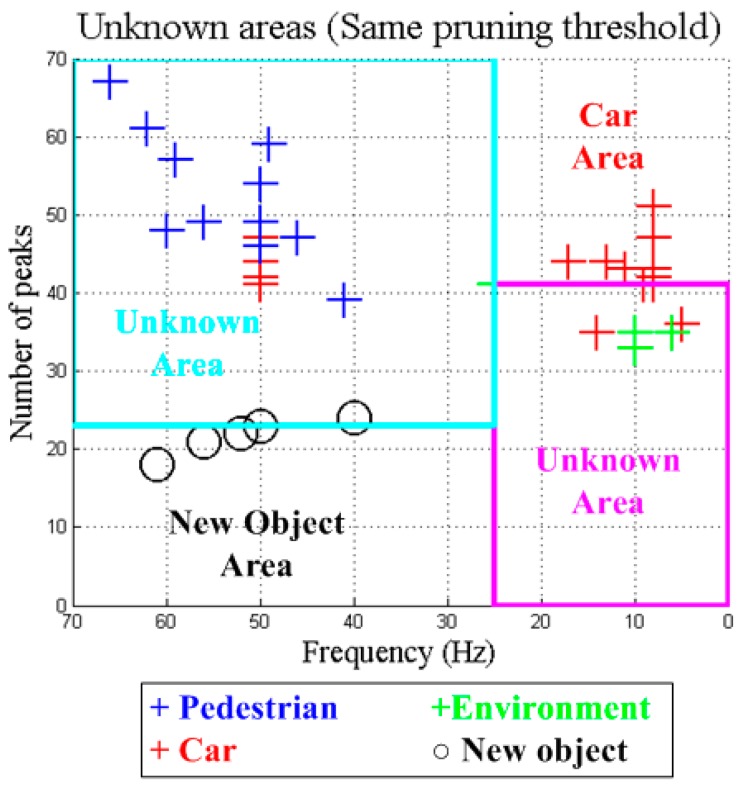
Unknown areas with the same pruning threshold (0.1).

In order to avoid the effect of the previous stage, the second one includes 10 instances of the same object in the training set, so there are more examples for a specific “unknown” object. However, results of this stage are very conditioned by the applied pruning threshold value. For instance, if the applied pruning threshold is the same like the one used in the previous decision tree, the new generated tree is able to classify the new object (Figure 17) including a “new object” category. However, it will create a wide area of unknown objects that collects those that were correctly classified in the previous decision tree (e.g., pedestrian). This implies that some early hits become new misses since pedestrian zone turns into “unknown” zone. Figure 18 shows the result with a pruning threshold of 0.1.

**Figure 19:**
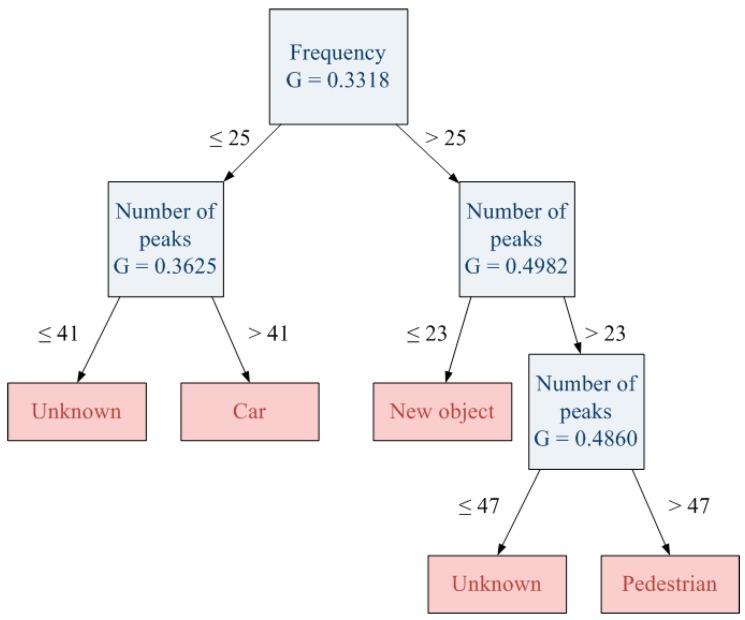
Decision tree with less pruning threshold (0.05).

**Figure 20:**
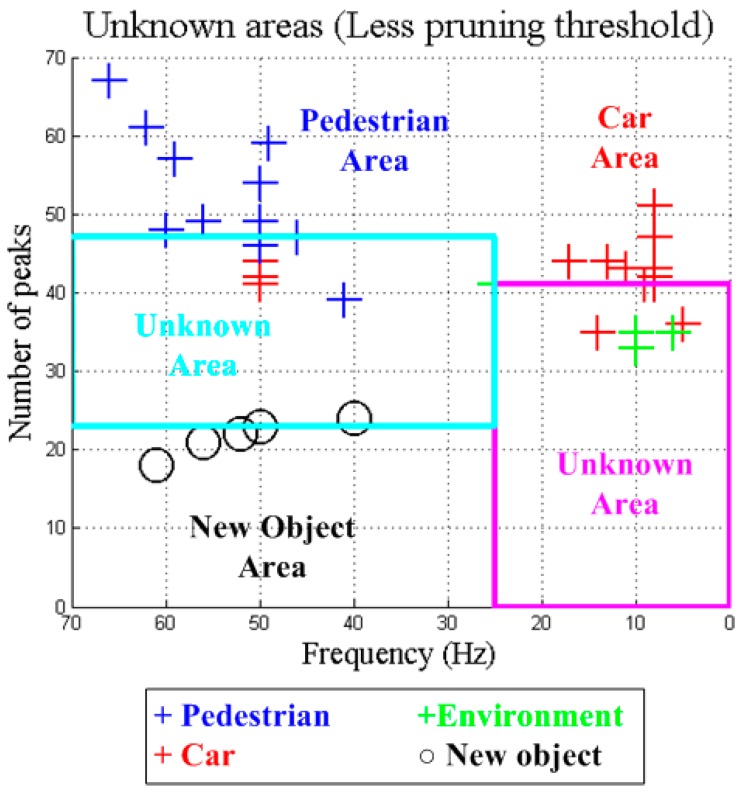
Unknown areas with the less prunning threshold (0.05).

In contrast, if the pruning threshold is reduced, the generated decision tree is able to classify both new and previously learned objects (Figure 19) and the area of the unknown objects will be more appropriate. Figure 20 shows the result for a half pruning threshold comparing with the original one (0.05). In this case, pedestrians are correctly classified and also new objects are detected.

### 4.3. System Performance and Memory Requirements

One of the main goals of this work is the implementation of the machine learning algorithms inside an embedded system; therefore the performance of this kind of platforms is very important. Therefore, both computing time and memory requirements are important factors to take into account for an embedded system.

#### 4.3.1. Computing Time Analysis

In this section the computing time spent in the creation of a decision tree using the modified C4.5 algorithm proposed in this work is analyzed.

**Figure 21:**
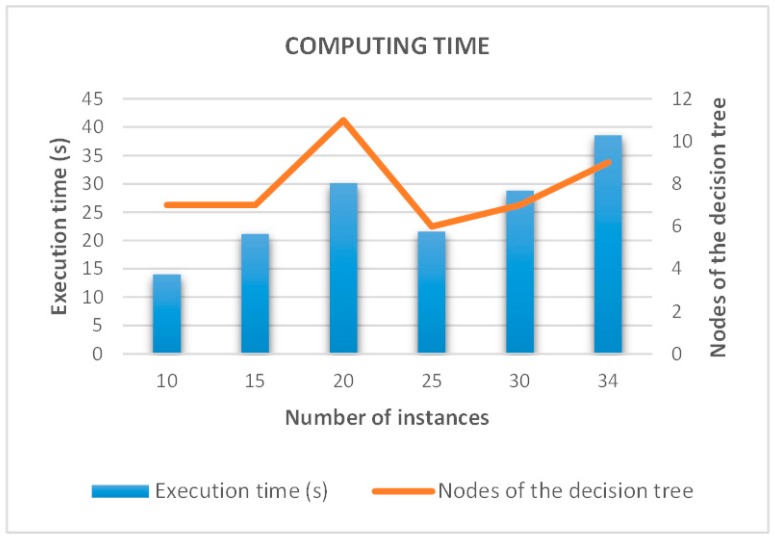
Computing time of the decision tree algorithm.

The computing time depends on both the number of instances of the training set and the number of nodes created in the decision tree (Table 3). Figure 21 shows the computing time against the number of instances of the training set. In general, the greater number of instances present in the training set, the longer computation time is required. However, in some cases the system deviates from the premise. As shown in Figure 21, in spite of increasing the number of instances from 20 to 25, the execution time is reduced. This is due to the fact that the number of nodes is also reduced in this case. Therefore, the decision tree is less complex and takes less time to create itself. 

**Table 3:** Computing time.

Computing Time		
Number of Instances	Execution Time (s)	Nodes of the Decision Tree
10	13.98	7
15	21.15	7
20	30.10	11
25	21.58	6
30	28.79	7
34	38.58	9

#### 4.3.2. Memory Requirements

**Figure 22:**
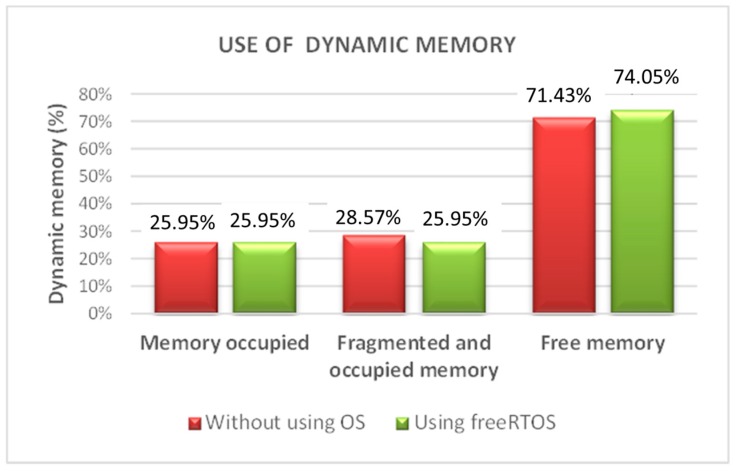
Dynamic memory using an OS.

As mentioned above, the system is implemented using a DSP (dsPIC33FJ64MC802) therefore a very limited memory size is available. The C4.5 algorithm makes use of recursion which causes that the dynamic memory used by the system becomes fragmented. This fragmentation leads to an inefficient memory use. Therefore, a good way to overcome this drawback is to use the memory management capabilities of an Operating System (OS). However, a complete OS has a strong footprint over the DSP program memory and for this reason, only the memory management layer of an OS is implemented. Table 4 and Figure 22 show the dynamic memory used by the system using or not the memory management layer of freeRTOS.

**Table 4:** Use of dynamic memory.

	Use of Dynamic Memory	
	Without Using OS	Using Free RTOS
**Free Memory**	71.43%	74.05%
**Memory Occupied**	25.95%	25.95%
**Fragmented Memory**	2.62%	0.00%

**Figure 23:**
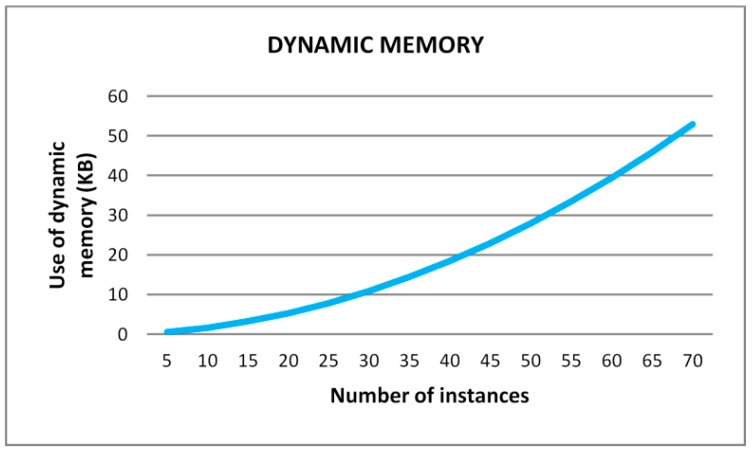
Evolution of the dynamic memory with the number of instances.

As it is shown in Table 4, using the memory management capabilities of the OS, the free dynamic memory increases by 2.62% with a footprint of just 780 Bytes over the data memory and 264 Bytes over the program memory. The required amount of dynamic memory for the learning architecture follows a geometric progression with the number of instances of the training set as shown Figure 23. For instance, it means that using the memory management capabilities of the OS and for a number of instances around 70, the saved memory will be 1310 Bytes.

## 5. Conclusions and Future Work

Taking into account the current simulation results, several conclusions are obtained considering different parts of the proposed scheme. On the one hand, for the decision tree algorithm, it is necessary to select the pruning threshold properly in order to optimize the “unknown” area. A low pruning threshold wastes a lot of space in the features space whereas a high pruning threshold degenerates the learning which causes the extinction of some classes (Figure 13). The optimization of the pruning threshold is mandatory due to the fact that learning capabilities increase when the “unknown” area is optimized. 

Something similar occurs with clustering threshold. The selection of an adequate threshold affects largely the number and the definition of new objects that the system is able to detect. A low clustering threshold implies the detection of too many objects that in fact belong to the same category. In contrast, a high clustering threshold produces the detection of objects which are poorly defined (Figure 14). Therefore, an appropriate selection of the clustering threshold may help the learning process since the system could learn homogenous objects that are properly defined. 

The presented results also demonstrate that the system, using the complete learning architecture, is able to learn new objects. However, it is unable to classify them as long as a high number of instances are learned. This effect is due to the fact that the number of learned instances for one object has to be comparable to the number of instances of the rest of the trained objects. Otherwise, the decision tree algorithm will assume that the object is a strange object and it will be classified as “Unknown”. Furthermore, in order to allow the system to evolve and to learn correctly, it is mandatory to adjust the pruning threshold. This adjustment could be automatic taking into account the number of instances of the training set and the number of different objects to classify. 

On the other hand, the proposed learning architecture, with the modified machine learning algorithms, is suitable for embedded systems in which memory and computational complexity are resources to be optimized. However, the required memory to execute the decision tree algorithm increases exponentially with the number of instances of the decision tree (Figure 23), which means that the size of the training set has to be bounded unless an external memory is included in the embedded system.

Summing up, early results demonstrate that the implementation of an adaptive-dynamic classification tree is possible. However, memory requirements have to be considered especially for embedded system developments. Moreover, it is important to mention that the system is not complete yet, since cooperative algorithms are not included although several cooperative algorithms have been studied independently [15,16]. For this reason the current system is able to learn new kind of objects but it is not able to label them. Therefore, future works are focused on improving the system including some cooperative algorithms for network implementation. In this way, the system behavior will be more adaptive and also more reliable since it could take advantage of the cooperation benefits. 
